# Efficacy of early endoscopy and colonoscopy in very elderly patients with gastrointestinal bleeding

**DOI:** 10.12669/pjms.331.11616

**Published:** 2017

**Authors:** Mustafa Celik

**Affiliations:** Mustafa Celik, Department of Gastroenterology, Pamukkale University Training and Research Hospital, Denizli; Turkey

**Keywords:** Endoscopy, Colonoscopy, very elderly patients, GIS bleeding

## Abstract

**Objective:**

We aimed to determine the efficacy and safety of early (within the first 24 hour from application) endoscopy and colonoscopy in very elderly patients with GIS bleeding.

**Methods:**

In this study, 95 patients were included who underwent early endoscopy with the pre-diagnosis of upper GIS bleeding or endoscopy-colonoscopy with the pre-diagnosis of lower GIS bleeding between 2012 and 2016. Endoscopy and colonoscopy procedures were compared in terms of the development of complications, tolerance of procedure, detection of bleeding site, and rate of therapeutic interventions performed for bleeding. In addition, the adequacy of colonoscopy preparation was evaluated.

**Results:**

There was no significant difference between endoscopy and colonoscopy on procedural complication (2.1% vs 2.8%) and tolerance rates (81% vs 74.2), (p>0.05). The bleeding site was detected during endoscopy in 34(56.6%) patients, and an endoscopic intervention was required for 15(25%) of these patients. The bleeding site was detected during colonoscopy in 12(34.3%) patients, and an endoscopic intervention was performed for two (5.7%) patients (p<0.05). In addition, the colonoscopy procedure was suboptimal in 26 of 35 patients (74.2%) because of poor preparations.

**Conclusion:**

Early endoscopy and colonoscopy are safe and well tolerated in very elderly patients with GIS bleeding. Upper GIS endoscopy in this patient population enables the detection of the bleeding site and an endoscopic intervention for the bleeding. However, colonoscopy is insufficient for detecting bleeding sites, and colonoscopic treatment of bleeding sites is difficult because of poor or no preparation in this patient population.

## INTRODUCTION

According to the 2010 World Health Organization (WHO) report, life expectancy has increased in the United States and almost worldwide. WHO defines elderly people as those aged ≥65 years and very elderly as those aged ≥80 years.

The use of gastrointestinal endoscopy in geriatric patients is increasing as a larger proportion of the population is reaching an advanced age. Upper endoscopy in elderly patients often provides diagnostic information that affects clinical therapeutic decisions.^1^-^3^ In this population, colonoscopy is mostly used for colorectal cancer screening and surveillance.^4^

Multiple studies have demonstrated the safety of upper endoscopy in elderly patients.^5^-^7^ However, the safety of colonoscopy remains unclear.^8^ Although colonoscopy is generally considered safe for elderly patients, advanced age is a risk factor for procedure-related adverse events.^9^ More than 1% of people aged ≥80 years are hospitalized each year because of gastrointestinal system (GIS) bleeding.^10^

The efficacy of upper endoscopy in GIS bleeding (74%) is significant in patients aged >50 years.^11^ However, colonoscopy in elderly patients may be more difficult in terms of GIS bleeding because they are more likely to have poor preparation than younger patients. The importance of adequate colonoscopy preparation cannot be overstated because poor preparation may result in missed lesions, procedure failure, prolonged procedure time, and increased procedural complications.^12^

We aimed to determine the efficacy and safety of early (within the first 24h from application) endoscopy and colonoscopy in very elderly patients with GIS bleeding.

## METHOD

We evaluated patients aged>80 years who presented to the emergency service and for whom gastroenterology consultation was requested based on a pre-diagnosis of GIS bleeding. In total, 95 patients were included who underwent early endoscopy with the pre-diagnosis of upper GIS bleeding or colonoscopy with the pre-diagnosis of lower GIS bleeding between 2012 and 2016.

We recorded hemoglobin (Hb), hematocrit (Htc), and blood urea nitrogen/creatinine (BUN/Cre) values, which were detected in tests performed inthe emergency room. Furthermore, patients’ use of acetylsalicylic acid (ASA), clopidogrel, coumadin, and nonsteroidal anti-inflammatory drugs (NSAIDs) was recorded.

Endoscopy and colonoscopy reports of patients were obtained from the hospital’s information processing and recording system. Endoscopy reports were examined, and the development of procedural complications and procedural tolerance were recorded. In addition, for patients who underwent endoscopy with a pre-diagnosis of upper GIS bleeding only, we recorded whether the bleeding site was detected or whether any endoscopic interventions for bleeding were performed.

Patients’ colonoscopy reports were examined, and the development of procedural complications and procedural tolerance were recorded. We also recorded whether the bleeding site was detected or whether any colonoscopic interventions for bleeding were performed. In addition, the adequacy of colonoscopy preparation was evaluated.

Endoscopy and colonoscopy procedures were compared in terms of the development of complications, tolerance of procedure, detection of bleeding site, and rate of therapeutic interventions performed for bleeding.

In patients for whom the reason for bleeding was determined, reasons for upper and lower GIS bleeding were evaluated. The study protocol was approved by the Non-invasive Clinical Research Ethics Committee of the University.

### Statistical analysis

Descriptive statistics were used for the comparison of datas. Chi-square test was used to compare proportions for normally distributed data. A P value <0.05 was considered statistically significant. All analyses were performed using SPSS 17.0 version.

## RESULTS

In total, 60 patients who underwent early endoscopy with the pre-diagnosis of upper GIS bleeding and 35 patients who underwent endoscopy and colonoscopy together with the pre-diagnosis of lower GIS bleeding were identified. The average age of patients was 84.42(±) 3.48 years. Fifty-two (54.7%) patients were males and 43(45.3%) were females. The average Hb value was 9.05(±)2.49 g/dL, and the average Htc value was 28.72(±)7.07. A BUN/Cre ratio of >20 was found in 81(85.2%) patients; that is, these patients had prerenal azotemia. Moreover, 60(63.1%) patients were using at least one medication, including NSAIDs, ASA, clopidogrel, and coumadin (Table-I).

**Table-I:** Age, sex, prerenal azotemia, average hemoglobin and hematocrit values, and anticoagulant–antiaggregant use in patients.

Age (years)	Sex (male)	Hgb (g/dL)	Htc (%)	BUN/Cre ratio (>20)	ASA, clopidogrel, NSAIDs,or coumadin Use (one of these)
84.42(±)3.48	52 (54.7%)	9.05(±)2.49	28.72(±)7.07	81/95(85.2)	60/95(63.1%)

When the 95 patients who underwent endoscopy were evaluated for procedural complications and tolerance, two (2.1%) were found to have developed hypoxia during the procedure and 77(81%) were able to tolerate the procedure without sedation (Table-II).

**Table-II:** Tolerance and complication rates of early endoscopy and colonoscopy in very elderly patients with gastrointestinal system bleeding.

	Endoscopy (n=95)	Colonoscopy (n=35)	p value
Tolerance	77(81%)	26 (74.2%)	p>0.05
Complication	2(2.1%)	1(2.8%)	p>0.05

The analysis of procedural complications and tolerance for the 35 patients who underwent colonoscopy revealed that one (2.8%) patient developed arrhythmia during the procedure and 26(74.2%) were able to tolerate the procedure without sedation. There were no statistically significant differences between endoscopy and colonoscopy in terms of the development of procedural complications and procedural tolerance (p>0.05) (Table-II).

Patients who underwent endoscopy with the pre-diagnosis of upper GIS bleeding were evaluated in terms of the detection of the bleeding site and any endoscopic interventions for bleeding. The bleeding site was detected during endoscopy in 34 of 60 patients (56.6%), and an endoscopic intervention was required for 15(25%) of these patients (Table-III).

**Table-III:** Detection of the bleeding site and therapeutic intervention rates of early endoscopy and colonoscopy in very elderly patients with gastrointestinal system bleeding.

	Endoscopy (n=60)	Colonoscopy (n=35)	p value
Detection of the bleeding site	34(56.6%)	12 (34.3%)	p<0.05
Therapeutic intervention	15(25%)	2(5.7%)	p<0.05

The patients who underwent colonoscopy were also evaluated in terms of the detection of the bleeding site and a colonoscopic intervention for the bleeding. The bleeding site was detected during colonoscopy in 12(34.3%) patients, and an endoscopic intervention was performed for two (5.7%) patients. The rates of detecting the bleeding site and performing therapeutic interventions were statistically significantly higher in endoscopy than in colonoscopy (p<0.05) (Table-III). In addition, the colonoscopy procedure was suboptimal in 26 of 35 patients (74.2%) because of poor preparations.

The most common cause for bleeding in patients who underwent colonoscopy with the pre-diagnosis of upper GIS bleeding was duodenum ulcer (17/28 patients, 60.7%). The reason for bleeding was identified as diverticulosis in eight of 12 patients (66.6%) who underwent colonoscopy because of lower GIS bleeding and in whom the bleeding site was detected. Angiodysplasia was observed in only one patient.

## DISCUSSION

Endoscopy procedures are vital for diagnosing and treating elderly patients. However, some complications are more common in this population; therefore, it is important to carefully evaluate the risks and benefits of these procedures in elderly patients.^8^ Upper endoscopy is the diagnostic modality of choice for acute upper GI bleeding,^13^,^14^ whereas colonoscopy is the standard diagnostic modality for acute lower GI bleeding.^15^,^16^

One of the main risks of performing endoscopy in elderly patients is the sedation used during the procedure. Previous studies suggested that there is an increased risk for elderly patients undergoing procedural sedation compared with younger patients.^5^,^17^,^18^ An alternative to procedural sedation is to perform unsedated endoscopy. Compared with younger patients, elderly patients may have a better tolerance for undergoing upper endoscopy or colonoscopy with little or no sedation.^19^ Our study showed that very elderly patients could tolerate early endoscopy and colonoscopy procedures performed following a pre-diagnosis of GIS bleeding without sedation. We also found that there was no significant difference between endoscopy and colonoscopy in terms of procedural tolerance.

Procedural success and morbidity in upper endoscopy in the elderly population is similar to those in the general population.^5^ Complications are reported in <2% of colonoscopies performed for lower GIS bleeding.^20^ We found that early upper endoscopy and colonoscopy had very low complication rates in very elderly patients with GIS bleeding, and there was no mortality because of procedural complications.

Once an upper GIS bleeding source is excluded, colonoscopy is the examination of choice for diagnosing and treating of acute lower GIS bleeding.^16^ Therefore, we performed upper endoscopy for our patients before colonoscopy, to exclude upper GIS bleeding. Peptic ulcer disease is the most common cause of upper GIS bleeding, including in elderly patients.^21^,^22^ We found that early upper endoscopy showed moderate efficacy for detecting GIS bleeding (56.6%) in patients aged >80 years. Duodenal ulcer is another common cause of upper GIS bleeding in very elderly patients. However, therapeutic interventions for active bleeding were performed in few (15%) patients.

The advantages of colonoscopy compared with other tests for lower GIS bleeding include its potential to precisely localize the bleeding site regardless of the etiology or rate of bleeding, the ability to collect pathologic specimens, and the potential for therapeutic intervention.^23^-^25^ The disadvantages of colonoscopy include the need for bowel preparationand poor visualization in an unprepared or poorly prepared colon in a patient with acute bleeding.^20^

We found that most of our patients (74.2%) had poor colonoscopy preparation. We identified the reason for bleeding in 34.3% of very elderly patients with lower GIS bleeding and performed therapeutic interventions in 5.7% of these patients. Also we assigned that endoscopy is superior than colonoscopy in identification of bleeding site and therapeutic intervention rates. In light of these data, poor colonoscopy preparation is the most determinative factor in diagnosing and treating lower GIS bleeding in very elderly patients.

In elderly patients, the most frequent causes of GIS bleeding are diverticulosis and angiodysplasia, and diagnosis for both is generally made by colonoscopy.^16^,^26^ However, we found that diverticulosis was the most common cause of lower GIS bleeding in our very elderly population (66.6%) but we found only one patient with angiodysplasia. This result might also be explained by poor colonoscopy preparations.

With respect to diverticular bleeding, blood and clots may be observed in numerous nonbleeding diverticula, making the identification of the bleeding diverticulum difficult. Therefore, we performed colonoscopy for only two patients. This may be afurther restriction on the use of colonoscopy in very elderly patients with lower GIS bleeding.

## CONCLUSION

Early endoscopy and colonoscopy are safe and well tolerated in very elderly patients with GIS bleeding. Upper GIS endoscopy in this patient population enables the detection of the bleeding site and an endoscopic intervention for the bleeding. However, colonoscopy is insufficient for detecting bleeding sites, and colonoscopic treatment of bleeding sites is difficult because of poor or no preparation in this patient population. Therefore, it is recommended that these procedures in this patient population should be performed in the most optimal conditions and only after sufficient preparation.
